# Preoperative Fasting Abbreviation and its Effects on Postoperative Nausea and Vomiting Incidence in Gynecological Surgery Patients

**DOI:** 10.1055/s-0040-1712994

**Published:** 2020-06-19

**Authors:** Gisele Vissoci Marquini, Francisco Edes da Silva Pinheiro, Alfredo Urbano da Costa Vieira, Rogério Melo da Costa Pinto, Maria Gabriela Baumgarten Kuster Uyeda, Manoel João Batista Castello Girão, Marair Gracio Ferreira Sartori

**Affiliations:** 1 Department of Gynecology, Escola Paulista de Medicina, Universidade Federal de São Paulo, São Paulo, SP, Brazil; 2 Department of Gynecology, Hospital Municipal Maternidade Dr. Odelmo Leão Carneiro, Uberlândia, MG, Brazil; 3 Faculty of Mathematics, Universidade Federal de Uberlândia, Uberlândia, MG, Brazil

**Keywords:** preoperative care, surgical procedures in gynecology, postoperative nausea and vomiting, carbohydrates, proteins, controlled random clinical trial, cuidados pré-operatórios, procedimentos cirúrgicos em ginecologia, náusea e vômito pós-operatório, carboidratos, proteínas, ensaio clínico controlado aleatório

## Abstract

**Objective** To investigate the effects of preoperative fasting abbreviation with a carbohydrate and protein-enriched solution, on postoperative nausea and vomiting (PONV) incidence in gynecological surgery patients, a population naturally at risk for such unpleasant episodes.

**Methods** The present prospective double-blind randomized study was performed at The Hospital Municipal e Maternidade Dr. Odelmo Leão Carneiro (HMMOLC, in the Portuguese acronym), in Uberlândia, state of Minas Gerais, Brazil, in partnership with the Gynecology Department of the Universidade Federal de São Paulo (UNIFESP), approved by the Human Research Ethics Committee of UNIFESP and the board of HMMOLC, and included in the Brazil Platform and in the Brazilian Clinical Trial Registry. After signing the consent form, 80 women, who were submitted to gynecological surgery in the period from January to June 2016, were randomized into 2 groups: control group ( *n* = 42) and juice group ( *n* = 38). They received, respectively, 200 mL of inert solution or liquid enriched with carbohydrate and protein 4 hours presurgery. The incidence, frequency and intensity of PONV were studied using the Visual Analogue Scale (VAS), with statistical analysis performed by the software IBM SPSS Statistics for Windows, Version 20.0 (IBM Corp, Armonk, NY, USA).

**Results** The incidence of nausea and vomiting was lower than in the literature, to this population, with 18.9% (14/74) for the control group and 10.8% (8/74) for the juice group, respectively, with no statistically significant difference between the groups.

**Conclusion** The incidence of nausea and vomiting was lower than in the literature, but it cannot be said that this is due to the abbreviation of fasting. It can provide greater comfort, with the possibility of PONV prevention in patients at risk for these episodes.

## Resumo

**Objetivo** Investigar os efeitos da abreviação do jejum pré-operatório, por meio de solução enriquecida com carboidrato e proteína, na incidência de náuseas e vômitos no pós-operatório (NVPO) em cirurgias ginecológicas, população naturalmente de risco para esses episódios desagradáveis.

**Métodos** O presente estudo prospectivo randomizado duplo-cego foi realizado no Hospital e Maternidade Municipal Dr. Odelmo Leão Carneiro (HMMOLC), em Uberlândia, estado de Minas Gerais, Brasil, em parceria com o Departamento de Ginecologia da Universidade Federal de São Paulo (UNIFESP), aprovado pelo Comitê de Ética em Pesquisa da UNIFESP, pela diretoria do HMMOLC, registrado na Plataforma Brasil e na plataforma de Registro Brasileiro de Ensaios Clínicos. Após assinatura do termo de consentimento, foram randomizadas 80 mulheres que se submeteram à cirurgia ginecológica, de janeiro a julho de 2016, em 2 grupos: grupo controle ( *n* = 42) e grupo suco ( *n* = 38) que receberam, respectivamente, 200 ml de solução inerte ou 200 ml de líquido contendo carboidrato e proteína, 4 horas pré-cirurgia. Foram estudadas incidência, frequência e intensidade de NVPO por meio da Escala Visual Analógica (EVA), com análise estatística realizada pelo programa IBM SPSS Statistics for Windows, Versão 20.0 (IBM Corp, Armonk, NY, EUA).

**Resultados** A incidência de náuseas e vômitos foi mais baixa que a esperada na literatura para essa população, com 18,9% (14/74) para o grupo controle e 10,8% (8/74) para o grupo suco, respectivamente, sem diferença estatisticamente significante entre os grupos.

**Conclusão** A incidência de náuseas e vômitos foi mais baixa do que na literatura; entretanto, não se pode afirmar que foi devido à abreviação de jejum. Essa medida pode trazer maior conforto com a possibilidade de prevenção de NVPO em pacientes de risco para esses eventos.

## Introduction

Prevention of postoperative nausea and vomiting (PONV) is recommended by perioperative care protocols, such as Enhanced Recovery After Surgery (ERAS) in Europe and Total Postoperative Recovery Acceleration (ACERTO, in the Portuguese acronym) in Latin America and Brazil, among others, as they are some of the most unpleasant postoperative symptoms. They are also considered to be the events that can most affect postoperative satisfaction and recovery. 1 2 According to the mentioned perioperative care protocols, the ingestion of a carbohydrate- and protein-enriched liquid up to 2 hours before surgery may also decrease the incidence of nausea and vomiting. 1 2

Given the clinical relevance of such perioperative episodes, Apfel et al 3 developed a risk score for PONV that was used for managing antiemetic prophylaxis. The independent risk factors for PONV identified by those authors were: female gender, nonsmoking patients, history of PONV and perioperative use of opioids. Based on the mentioned criteria, those authors developed a low-, medium- and high-risk classification system that allows to estimate the possibility of PONV in patients. Each criterion receives a point, and the high-risk patients are those with scores > 3 points. 3 4

Many perioperative care routines recommend controlling anxiety, nausea and vomiting as a way of humanizing hospital care. 5 6 However, little is known about preoperative fasting abbreviation as a tool to relieve tension and suffering of women undergoing gynecological surgery, who are naturally at risk for nausea and vomiting according to the criteria by Apfel et al. 3 4 This simple measure could reduce such negative postoperative symptoms in gynecological surgeries.

Toward this scenario, the present study aims to evaluate the incidence, frequency and intensity of PONV in gynecological surgery patients who abbreviated preoperative fasting, one of the main recommendations of the ERAS and ACERTO protocols.

## Methods

This was a prospective, randomized, parallel group study (enrollment, intervention allocation, follow-up and data analysis) for preoperative fasting abbreviation in gynecological surgeries. It was performed at The Hospital Municipal e Maternidade Dr. Odelmo Leão Carneiro (HMMOLC, in the Portuguese acronym), a general hospital of medium complexity, located in Uberlândia, state of Minas Gerais, Brazil, in partnership with the Universidade Federal de São Paulo (UNIFESP, in the Portuguese acronym).

The present study was submitted to the board of the HMMOLC and the Human Research Ethics Committee of the UNIFESP and obtained approval under the opinion number 1.192.130, under the Certificate of Presentation for Ethical Consideration (CAAE) number 48103015.8.0000.5505. It was included in the Brazilian Clinical Trial Registry (ReBec, in the Portuguese acronym) of the WHO International Clinical Trials Registry Platform ( http://www.who.int/ictrp/en/ ), under the register code RBR-66 gqfs.

The participants of the present study were patients with indication of gynecological surgery, from January to June 2016 at the HMMOLC. The eligibility criteria were: age between 18 and 70 years old, preanesthetic evaluation, American Society of Anesthesiologists (ASA) score I or II, body mass index (BMI) ≤ 40 kg/m2. Patients with infections, gastroesophageal reflux, use of steroids for at least 6 months prior to surgery, renal or hepatic disease, c-reactive protein > 6 mg/dl, diabetes mellitus and a surgery lasting ≥ 4 hours were excluded.

After recruiting the patients, they were randomized into two groups. The method and mechanism used to generate the random allocation sequence was developed by Microsoft Excel 2014 (Microsoft Corporation, Redmond, WA, USA).

One of the co-authors generated the random allocation sequence (Pinto R. M. C.), Vieira A. U. C. enrolled the participants and another coauthor (Pinheiro F. E. S.) assigned participants to interventions. The blinding was done and the first cited author (Marquini G. V.) was blinded after assignment to interventions.

The groups were: control group and juice group, which received respectively 200 mL of inert solution (composed by distilled water, 4 drops of red dye and 2 drops of sucrose-based sweetener) or clarified supplement rich in carbohydrate (89%) and whey protein (11%) produced by a pharmaceutical industry, offered to patients without a packaging label. They were instructed to ingest the received fluid 4 hours before surgery.

The authors determined a 4-hour period before surgery instead of 2, as endorsed by anesthesiology societies, in reference to the paradigm changes that are better adhered to gradually. Despite the fact that the ERAS protocol and anesthesiology societies release fluid intake up to 2 hours before surgery, 7 in practice, adherence to these recommendations may have resistance, since preoperative fasting averages in Brazil are ∼ 16 hours according to the BIGFAST multicenter study. 8 For these reasons, it was decided to start introducing 4-hour fast abbreviation recommendations into practice as a way to ensure the fearless adherence of the surgeons.

Likewise, as a measure of gradual paradigm shifting to favor adherence to ERAS, the authors maintained the recommendation in practice of the local hospital for the 12-hour fasting study for solids, despite the recommendation of anesthesiology societies to support the 6-hour period.

The authors believe that this respect to traditional paradigms did not influence the objective of the present study, since the objective was not the evaluation of fasting recommendations for solids, but the evaluation of nausea and vomiting with abbreviated liquid fasting.

The authors can state that the intervention effectively happened once the patients were hospitalized the night before the surgery and the fluid was ingested under the supervision of the nursing team.

After group randomization, the fluid to be ingested was given, with written instructions on the time of ingestion, hospitalization and fasting for solids 12 hours before surgery, as previously mentioned, following the preanesthetic recommendation of the hospital service.

The following clinical parameters were analyzed: age, weight, height, body mass index (BMI), hospitalization and discharge dates, previous PONV history, perioperative opioid use, smoking habits, and surgery start and end times.

To test the benefit hypothesis of preoperative fasting abbreviation, the incidence, frequency and intensity parameters of nausea and vomiting episodes were studied using the Visual Analogue Scale (VAS). The VAS is a line measuring 10 cm, with marks at both ends corresponding to “no nausea or vomiting” or “no symptoms” and “worst nausea or vomiting” or “worst symptoms.” All of the patients were asked to evaluate and mark on the line the nausea or vomiting symptom to quantify its intensity.

The incidence corresponded to the number of patients who had nausea and vomiting. The frequency was represented by the number of nausea or vomiting episodes (0–1 or 2–3 or more) in each patient. The nausea and vomiting intensity was classified as: mild if VAS < 3, moderate if in between 3 and 7, and intense if VAS > 7, and it was quantified in means according to the numbers marked in the VASs. The questionnaire with the VASs was applied by the researcher 10 hours after surgery, to wait the maximum time of metabolic reaction to the trauma, before the authorization of the gynecological surgeon to stop the fasting period (often not < 12 hours).

The sample size calculation to obtain an analytical sample for independent groups was performed using the formula described by Fontelles et al 9 ;

Where:

zα/2 is the alpha error value (two-tailed),

zβ is the beta error value,

s is the standard deviation,

*d* is the minimum difference to be detected.

The statistical analysis of this calculation was based on previous studies by Faria et al 10 and by Dock-Nascimento et al. 11 The sample size was determined for identification, with 95% confidence (error α = 0.05), a difference, if any, of at least 5 μU/mL between the means of insulin values of both evaluated groups. A total of 30 patients in each group were estimated to be sufficient, with 90% test power, predicting a 50% difference, if any, between the groups for nausea and vomiting in the VAS results.

The descriptive analysis was performed, presenting the frequency measurements in percentages for qualitative variables. The chi-squared test was used to verify the association between the groups and the qualitative variables (nausea and vomiting). The level of significance was 0.05. Statistical analyzes were performed using IBM SPSS Statistics for Windows, Version 20.0 (IBM Corp., Armonk, NY, USA).

### Sample

**Fig. 1:**
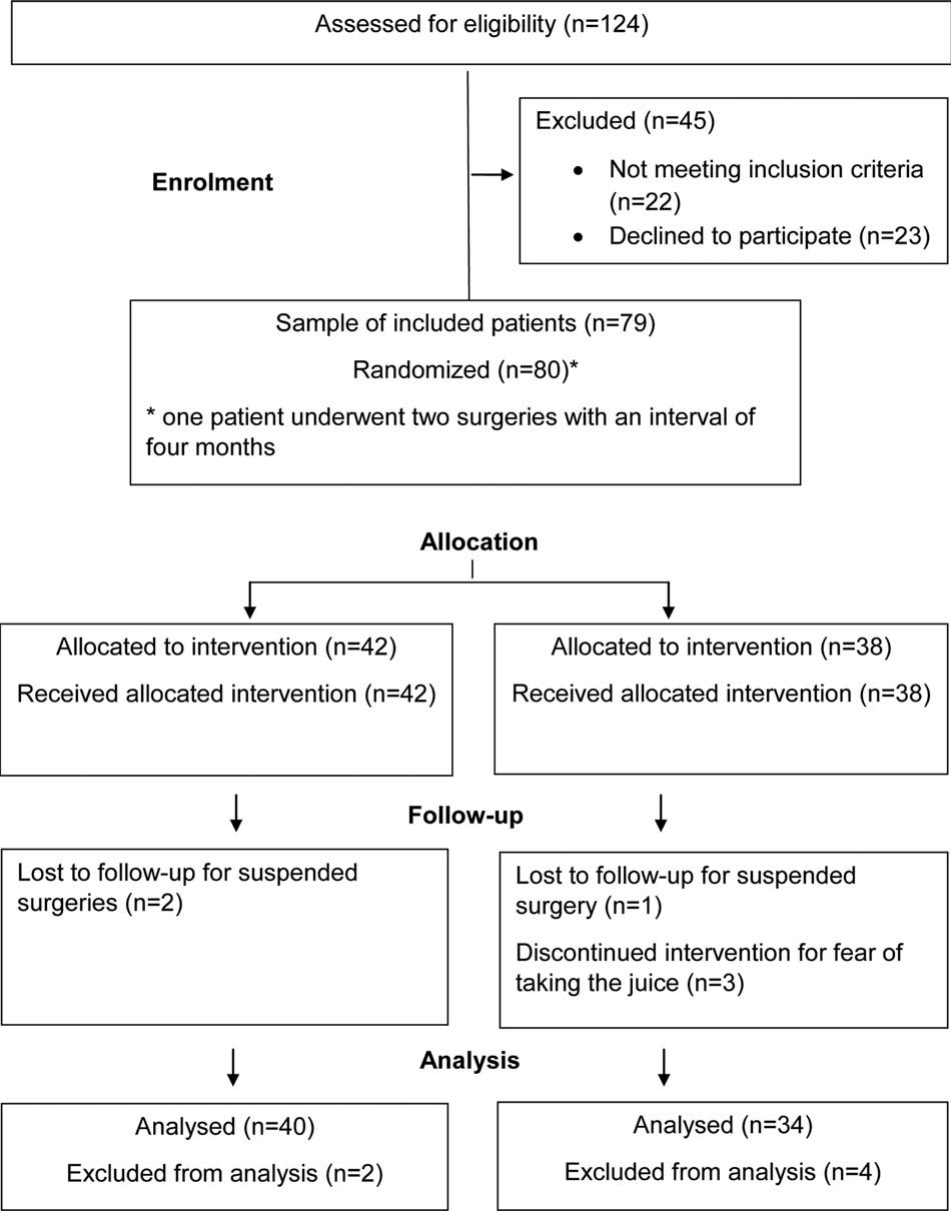
Study flow diagram of the progress through the phase of a two-group parallel randomized trial for preoperative fasting abbreviation in gynecological surgeries.

A total of 124 gynecological surgeries were scheduled at the HMMOLC from January 2016 to July 2016. Of the 124 patients scheduled, 23 did not agree to participate in the study and 22 were excluded or did not meet the eligibility criteria. The total number of (female) patients who started the study was 79, considering that one patient underwent two procedures and accepted inclusion in both hospitalizations. Therefore, the total number of randomized cases was 80, of which 42 were allocated in the placebo group and 38 in the juice group ( Fig. 1 ). Of these patients, as shown in the Study Flowchart ( Fig. 1 ), 74 completed the protocol (control group = 40 and juice group = 34). The six losses were due to surgery suspension ( *n* = 3) or fear of taking the juice ( *n* = 3).

Given the clinical relevance of criteria in nausea and vomiting evaluation by Apfel, 3 the present study characterized the sample according to those criteria. Tables 1 and 2 show that the sample was homogeneous. In addition, they demonstrate the sample distribution according to age, duration of the surgery (minutes) ( Table 1 ) the Apfel classification (risk factors for nausea and vomiting) and scoring criteria, such as female gender, smoking habits, vomiting history and perioperative opioid use ( Table 2 ).

**Table 1:** Sample distribution according to clinical variables

Risk factors for PONV		Total ( *n* = 74)	Control Group ( *n* = 40)	Juice Group ( *n* = 34)	Chi-square	*p* -value
No smoking habits		66 (89.1%)	36 (90%)	30 (89.3%)	0.5943	1
PONV history		7 (9.5%)	4 (10%)	3 (8.9%)	0.2745	0.7416
Perioperative use of opioids		54 (73.0%)	31 (77.5%)	23 (67.7%)	0.9046	0.4213
Incidence of nausea (number of patients)		14 (18.9%)	7 (17.5%)	7 (20.5%)		
Frequency of nausea (number of episodes)		2	1	1	0.6630	1
Intensity of nausea (VAS median)		4.8	5.2	4.5		
Incidence of vomiting (number of patients)		8 (10.8%)	4 (10.0%)	4 (11.7%)		
Frequency of vomiting (number of episodes)		2	1	1	0.5943	1
Intensity of vomiting (VAS median)		6.3	6.5	6.2		
Apfel Classification	Low- I	20 (27.1%)	10 (25.0%)	10 (29.5%)		
	Medium- II	40 (54%)	24 (60.0%)	16 (47.0%)	1.4085	0.4853
	High- III	14 (18.9%)	6 (15%)	8 (23.5%)		

Abbreviations: PONV, postoperative nausea and vomiting; VAS, visual analogue scale.

**Table 2:** Sample distribution according to risk factors for PONV

Variable	Group	Mean	CI (95%)	Mean	min-max	*p-value*
Age	Control	39.2	36.3–42.1	38.50	25–58	0.552
(years old)	Juice	42.5	38.3–43.4	42.4	22–64	
Duration of surgery	Control	71.4	52.2–90.5	50.0	20–180	0.886
(minutes)	Juice	77.9	56.8–99.0	60.0	47–132	

## Results

The data in Table 1 shows a paired sample between the groups according to age and time surgery.

According to data from Table 2 , there was a predominance of Apfel II among groups, that is, moderate risk for PONV. The predominant criteria in the Apfel score were: female gender and nonsmokers. Those who presented Apfel III had one more risk factor because they had received intraoperative opioid prescription, which was the third most frequent risk factor. The PONV history was the least contributing factor for the classification of risk for PONV.

The patients were not separated by type of surgery due to the homogeneity of the gynecological surgical practice of the study hospital. The surgical access studied was abdominal laparotomy, with surgical time of < 4 hours, as a study criteria. Within this context, the most performed surgeries were abdominal hysterectomies and tubal ligation without statistical difference between the groups.

There were no anesthetic complications, nor even aspirations of gastric contents during the procedure in the present sample. Table 2 presents data on the nausea analysis, including incidence, frequency and intensity of the episodes.

The nausea incidence in the present study was 18.9% (14/74). There was no statistically significant difference in the nausea episodes between groups, whether in incidence, frequency or intensity when comparing the intake of both the inert solution and the carbohydrate and protein-enriched liquid. No patient in the juice group developed severe nausea.

In addition, Table 2 presents data on the vomiting analysis, including incidence, frequency and intensity of the episodes. The vomiting incidence was 10.8% (8/74), also with no statistically significant difference between groups for the studied parameters.

Table 3 shows that among the patients who had PONV, all scored on the Apfel scale because they were female and received opioids throughout the perioperative period. The fact that they were predominantly nonsmokers also contributed for them to be at risk for PONV. The least scoring criterion was the previous history of nausea and vomiting.

**Table 3:** Frequency of APFEL criteria among the 14 patients with PONV

APFEL criteria	Frequency N (%)	Total N (%)
Female gender	14 (100)	14 (100)
Opioid use	14 (100)	14 (100)
Nonsmoker	12 (87.5)	14 (100)
PONV history	4 (28.5)	14 (100)

Abbreviation: PONV, postoperative nausea and vomiting.

## Discussion

Nausea and vomiting, as well as pain, are among the most undesirable postsurgical side effects. 4 10 11 12 13 14 15 16 It is important for the gynecological surgeon and team to determine the appropriate management of these unpleasant effects, with practices that allow greater comfort in the recovery of the patients. Dalila et al 4 and Odom-Forren et al 17 18 suggest evaluating PONV as a quality variable in postanesthetic recovery units (PARUs). 4 17 18

Based on the model proposed by Dalila et al, 4 a VAS for measuring PONV intensity, considered an accurate and reliable instrument for the evaluation of this parameter, was applied in the present study. According to the PONV VAS, a score > 70 mm was the cutoff point for nausea or severe vomiting. 4 17

The present study had a relatively low incidence of nausea and vomiting when compared with data from similar populations in the literature. Studies in low-risk populations for PONV indicate an incidence ranging from 20 to 30%. 19

However, in the present sample, moderate to high-risk patients for PONV were predominant according to the criteria by Apfel. The incidence can reach as high as 70% in populations of moderate to high risk. 19

This decrease in the estimated incidence for this sample may be associated with preoperative fasting abbreviation, since the samples were homogeneous between the groups with no intervention other than the ingestion of liquid in a shorter preoperative period.

The absence of statistical difference between the groups suggests that the liquid offered is not a determining factor. Fasting abbreviation performed either with a carbohydrate and protein-enriched liquid or with an inert solution, both clear liquids, in the volume tested, may positively impact PONV reduction.

The importance of this finding is that the reduction of PONV episodes, with simple measures such as preoperative fasting abbreviation, can reduce hospital costs and complication rates, such as dehydration, electrolyte imbalance, suture dehiscence, hemorrhage, rupture of the esophagus and airway compromise. 4

According to data from the present study, among the patients who presented PONV, all scored the Apfel criteria for being female and for receiving intraoperative opioid administration. Smoking was the patient-dependent factor that contributed most to PONV. 20 21 22 23 The present study suggested that, among the modifiable, patient-dependent factors, this one is the most striking factor in risk classification for PONV, according to the criteria by Apfel ( Table 3 ).

Smoking is an important public health problem, as well as a common cause of preventable death in the world. 20 21 Smoking is a risk factor for several perioperative adverse events. 22 23 24 Nonetheless, being an active smoker also protects against PONV. According to the criteria by Apfel , nonsmoking patients are considered at high risk of developing PONV. 3 25 26 27 28 29

The mechanism involved in reducing the risk of PONV in smokers is still unknown. It is not known whether it is directly related to cigarette smoke constituents; however, there are some possible mechanisms. Chronic exposure to cigarette smoke would be one of them, resulting in protection against PONV. At the onset of smoking, individuals often experience nausea due to the stimulation of nicotinic acetylcholine receptors, but with the chronic use of cigarettes they would develop tolerance against these receptors. 30 31

Smoking patients may still be cross-tolerant to other emetic stimuli, such as anesthesia and surgery. Chronic exposure to smoke also produces changes in microsomal liver enzymes that metabolize nicotine and other components of cigarette smoke. 10 30 31 This may affect the metabolism of drugs used in the preoperative period and the ability of these drugs to produce PONV. 10 30 31

According to the ERAS and ACERTO protocols, careful management of opioids may also minimize the risk for these unpleasant episodes. In the present study, all of the patients who had PONV received intraoperative opioids. The decision on whether to use opioids may be multifactorial, and its prescription is based on recommendation protocols and on the subjective decision and clinical criteria of the anesthesiology team.

Although the indication of opioid use in anesthesiology has its recommendation protocols, the clinical and individual criteria of anesthesiologists stand out in practice. According to data collected in the present study, it is possible to observe a preference among anesthesiologists to use opioids to alleviate the pain of the surgical patient, since even among patients without nausea and vomiting, almost all of the sample was prescribed.

Given the perspective of opioid administration and other risk factors for PONV that do not depend on the intervention of a gynecologist at the time of surgery, such as female gender, PONV history and smoking habits, data from the present study reinforce that the adherence to preoperative fasting abbreviation may be an essential window of opportunity for PONV prevention in gynecological surgeries. In addition to being recommended by protocols such as the ERAS and the ACERTO project, this measure represents a simple action for perioperative care practice.

## Conclusion

Carbohydrate and protein supplementation 4 hours before surgery does not improve the occurrence of nausea and vomiting, since there was no difference between the groups and the abbreviation for the control group was based on an inert liquid with no carbohydrate and protein supplementation. The occurrence of nausea and vomiting was lower than that found in the literature, but it cannot be said that this is due to the abbreviation of fasting, because there is no comparison of factors such as the type of surgery and the age of the patients in the present study with the published studies. Preoperative fasting abbreviation, recommended by societies that validate perioperative care, either by an inert liquid or by a carbohydrate and protein-enriched solution, can provide greater comfort, with the possibility of PONV prevention in patients at risk for these episodes.
